# Prior SARS‐CoV‐2 infection balances immune responses triggered by four EMA‐approved COVID‐19 vaccines: An observational study

**DOI:** 10.1002/ctm2.869

**Published:** 2022-05-11

**Authors:** Roberto Lozano‐Rodríguez, Verónica Terrón‐Arcos, Karla Montalbán‐Hernández, José Carlos Casalvilla‐Dueñas, Marta Bergón‐Gutierrez, Alejandro Pascual‐Iglesias, Jaime Valentín Quiroga, Luis A. Aguirre, Rebeca Pérez de Diego, Carmen Vela‐Olmo, Lissette López‐Morejón, Alejandro Martín‐Quirós, Álvaro del Balzo‐Castillo, María A. Peinado‐Quesada, Miguel A. García‐Garrido, Laura Gómez‐Lage, Carmen Herrero‐Benito, Irene Llorente‐Fernández, Gema Martín‐Miguel, Margarita Torrejón, Carolina Cubillos‐Zapata, Carlos del Fresno, José Avendaño‐Ortiz, Eduardo López‐Collazo

**Affiliations:** ^1^ The Innate Immune Response Group IdiPAZ La Paz University Hospital Madrid Spain; ^2^ Tumour Immunology Lab IdiPAZ La Paz University Hospital Madrid Spain; ^3^ Eurofins‐Ingenasa Madrid Spain; ^4^ Emergency Department and Emergent Pathology Research Group IdiPAZ La Paz University Hospital Madrid Spain; ^5^ Intensive Care Unit Hospital 12 de Octubre Madrid Spain; ^6^ Respiratory Diseases Group IdiPAZ La Paz University Hospital Madrid Spain; ^7^ Network Biomedical Research Center in Respiratory Diseases (CIBERES) Madrid Spain

Dear Editor,

We have studied humoral and cellular responses in a Spanish cohort of 433 volunteers immunized with four COVID‐19 vaccines (Ad26.CoV2.S, BNT162b2, ChAdOx1 and mRNA‐1273) approved by the European Medicines Agency (EMA), classified as naïve or recovered according to whether they were previously infected by SARS‐CoV‐2. Both humoral and cellular responses were higher in those naïve individuals immunized with mRNA‐type vaccines (mRNA‐1273 and BNT162b2) compared to those inoculated with viral‐vector vaccines (ChAdOx1 and Ad26.CoV2.S). These differential responses over time were significantly attenuated in COVID‐19‐recovered subjects, as in these conditions we did not find any differences between the four vaccination regimens, neither with one nor two doses of ChAdOx1 or BNT162b2 vaccines in these individuals.

We have recently reported that previous medical history of COVID‐19 differentially determines the functional B and T cell‐mediated responses to BNT162b2 vaccination over time.^1^ Herein, we have extended that study in a cohort of volunteers vaccinated with four regimens used in Spain. The volunteers were classified as either naïve or recovered according to whether they had been previously infected by the SARS‐CoV‐2 (Figure 1 and Supporting information Tables S1 and S2).

**FIGURE 1:**
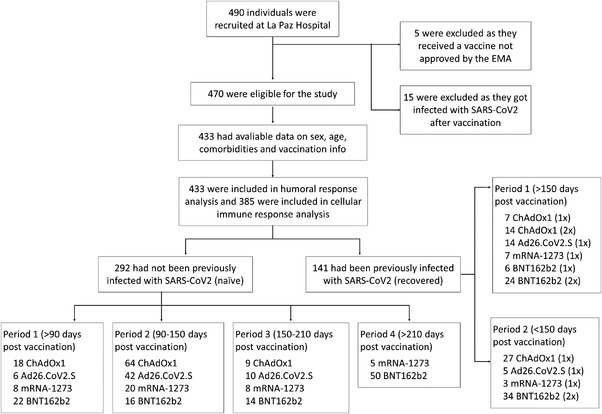
Study population cohorts. The population included individuals who were fully vaccinated before 8 August 2021. Recruitment was conducted from 21 February 2021 to 21 October 2021

Both BNT162b2 and mRNA‐1273 induced higher levels of anti‐Spike RBD IgG antibodies than viral‐vector vaccines ChAdOx1 and Ad26.CoV2.S, remaining higher at least until 150 days after vaccination (Figure 2A). In addition, mRNA‐based vaccines induced higher titers of neutralizing antibodies than viral‐vector vaccines, keeping for around 90–150 days (Figure 2B) after which, such differences were not observed. Additionally, mRNA‐based vaccines reached higher levels of anti‐Spike RBD IgA antibodies than viral‐vector vaccines only in the first 3 months post‐vaccination (<90 days) perceiving low levels 90–210 days post‐vaccination and without differences between all vaccination regimens (Supporting information Figure S1). Curiously, reduced levels of anti‐Spike RBD IgA at late points (>150 days after full vaccination) coincide with the neutralization capacity downregulation (Figures 2B and Supporting information Figure S1). Other authors have reported that in natural infection anti‐Spike RBD IgA antibodies showed to be the major responsible of the neutralizing ability in early stages.^2^ Regarding the cellular response, CD4^+^ but not CD8^+^ T‐cell activities against SARS‐CoV‐2 spike peptide pool (Supporting information Figure S2A) remained high at late‐time points in naïve individuals immunized with the mRNA‐type vaccines (Figures 2C and Supporting information Figure S2B). SARS‐CoV‐2 spike‐specific cytokines production also showed enhanced responses after vaccination with mRNA‐type vaccines (Supporting information Figure S3). Additionally, these antigen‐specific T‐cell responses were characterized by a high proportion of effector and central memory subpopulations, but with a light decrease in effector memory T cells and an increase in central memory T cells long after vaccination (Supporting information Figure S4).

**FIGURE 2:**
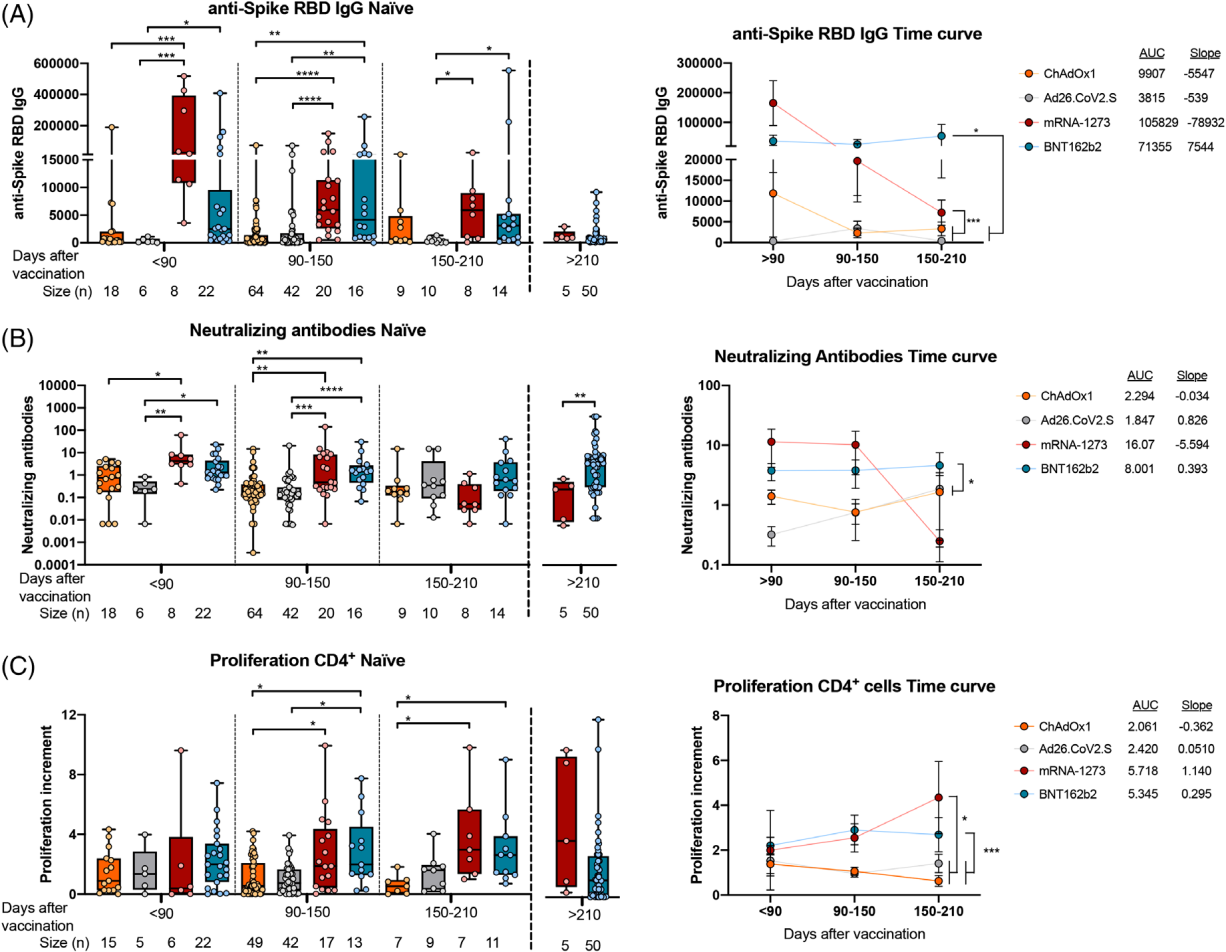
Humoral and cellular CD4^+^ T‐cell response triggered by ChAdOx1, Ad26.CoV2.S, mRNA‐1273 and BNT162b2 in naïve subjects. A total of 292 no‐previously infected (naïve) individuals from Spain were included in our study. They were classified according to their EMA vaccination regimen and the time since full vaccination. (A) The anti‐spike RBD IgG levels in relative units determined by enzyme‐linked immunosorbent assay in plasma from naïve individuals are shown. (B) The neutralizing antibodies titers (10^8^/[free ACE2] in log10 scale) determined by using a competitive immunoassay in plasma from naïve individuals are shown. (C) The proliferation increments of CD4^+^ cells after 5 days of stimulation of peripheral blood mononuclear cells from naïve individuals with SARS‐CoV‐2 peptide pool in vitro are shown. *p < .05; **p < .01; ***p < .001; ****p < .0001 in Kruskal–Wallis with Dunn's multiple post‐hoc test for group comparisons. Data in time curves are expressed as mean ± SEM; AUC, area under the curve; *p < .05; **p < .01; ***p < .001; ****p < .0001 in Tukey's multiple test for time curve comparisons

Differential responses observed in naïve individuals over time were attenuated in COVID‐19‐recovered subjects. We did not find patent differences in the immune responses between the four vaccination regimens in recovered individuals, neither one nor two doses of BNT162b2 or ChAdOx1 vaccines (Figure 3). Besides, when comparing humoral and cellular responses between naïve and COVID‐19‐recovered subjects, we found higher anti‐spike RBD IgG and neutralizing antibodies levels in recovered individuals, but no differences in the cellular CD4^+^ T‐cell response were found (Figure 4).

**FIGURE 3:**
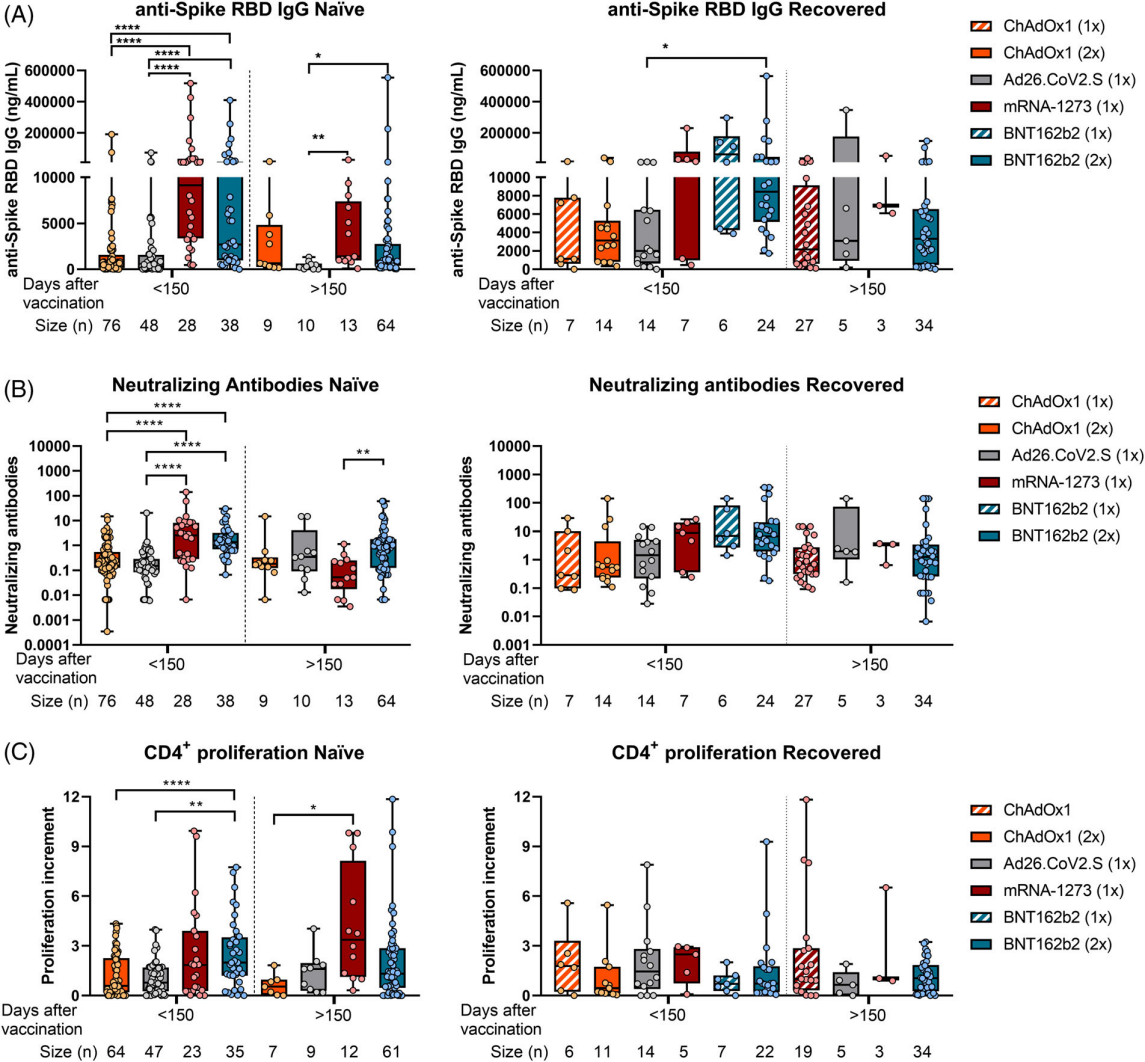
Humoral and cellular CD4^+^ T‐cell response triggered by ChAdOx1, Ad26.CoV2.S, mRNA‐1273 and BNT162b2 in naïve and COVID‐19‐recovered individuals over time. A total of 433 individuals from Spain were included in our study, 292 no‐previously infected (naïve) and 141 previously infected with SARS‐CoV‐2 (recovered). They were classified according to their EMA vaccination regimen and the time since full vaccination. (A) The anti‐spike RBD IgG levels, (B) neutralizing antibodies levels and (C) the SARS‐CoV‐2 specific CD4^+^ proliferation in naïve and COVID‐19‐recovered individuals for the different vaccination strategies are shown. *p < .05; **p < .01; ***p < .001; ****p < .0001 in Kruskal–Wallis with Dunn's multiple post‐hoc test for group comparisons

**FIGURE 4:**
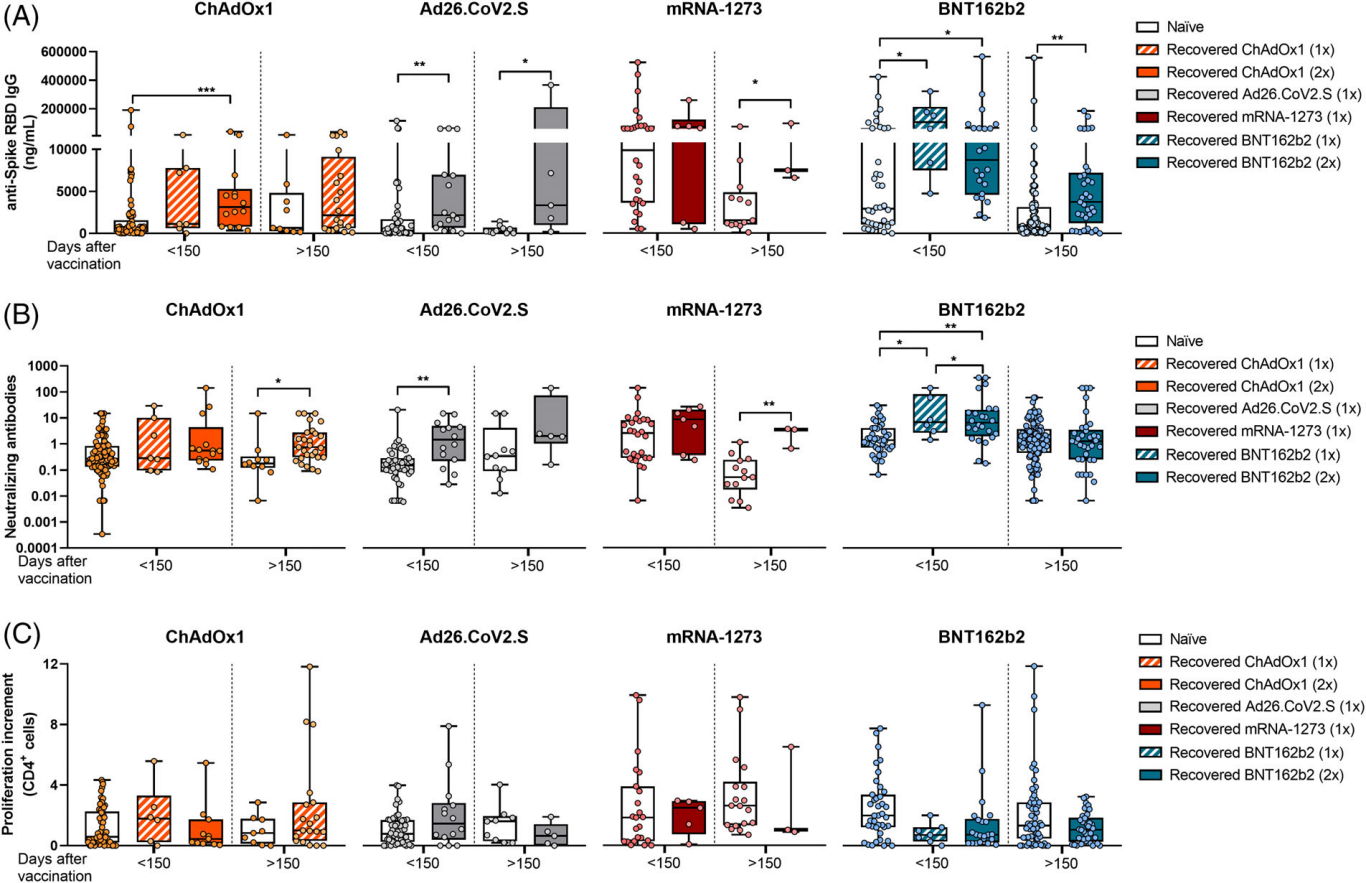
Comparison of SARS‐CoV‐2‐specific immune response between naïve and COVID‐19‐recovered individuals according to their vaccination regimen. (A) The comparison of plasmatic anti‐spike RBD IgG levels in naïve and COVID‐19‐recovered individuals for the different vaccination strategies is shown. (B) The comparison of plasmatic neutralizing antibodies levels in naïve and COVID‐19‐recovered individuals for the different vaccination strategies is shown. (C) The comparison of Spike SARS‐CoV‐2‐specific CD4^+^ proliferation in naïve and COVID‐19‐recovered individuals for the different vaccination strategies is shown. *p < .05; **p < .01; ***p < .001; ****p < .0001 in Kruskal–Wallis with Dunn's multiple post‐hoc test and Mann–Whitney test for three or two group comparisons, respectively

On 27 December 2020, the vaccination campaign began in Spain. One year later, this country had become one of the countries with the highest vaccination ranks, reaching a percentage of 80 of the population with a complete schedule. To date, the EMA has authorised five vaccines: ChAdOx1, Ad26.CoV2.S, mRNA‐1273, BNT162b2 and NVX‐CoV2373; however, the latter has not been administered in Spain. An extensive information on the prevention of hospitalization and mortality due to vaccination has been already assessed. Several studies performed in Spain confirm the impact of ratcheting up SARS‐CoV‐2 vaccination has had on declining COVID‐19 hospitalizations and lethality rates, even though a lack of efficiency reducing the transmission rate was observed.^3^, ^4^ Additionally, these high ranks of vaccination have supposed a reduction in the use of medical resources with a high social returns.^5^ Nevertheless, a comparison between the differential immune responses triggered by each vaccine is still lacking. This is especially grievous in the case of cellular responses, since most studies solely focus on the antibody production.^6^ Additionally, a medical history of COVID‐19 infection has usually been an exclusion criterion in most of the reported studies.

Our findings support the use of mRNA‐type vaccines for the induction of more robust humoral and cellular immune responses compared to viral‐vector vaccines. Epidemiological data indicate BNT162b2 and mRNA‐1273 vaccines have higher efficacy protecting against novel variants than the viral‐vector ChAdOx1 and Ad26.CoV2.S vaccines.^7^, ^8^ Additionally, previous history SARS‐CoV‐2, seem to have even higher efficacy.^9^, ^10^ Our findings on immune responses provide new evidence for explaining these data. A booster dose, especially in naïve individuals vaccinated with Ad26.CoV2.S or ChAdOx1, would be recommended in order to reach levels of both humoral and cellular responses similar to those observed in COVID‐19‐recovered subjects.

## Supporting information

Supporting Information

Supporting Information
