# Supplementary material for: Prior SARS‐CoV‐2 infection balances immune responses triggered by four EMA‐approved COVID‐19 vaccines: An observational study
Source: Clin Transl Med. 2022 May 11;12(5):e869. doi: 10.1002/ctm2.869 (PMC9092002; doi:10.1002/ctm2.869)
Supplement: Supplementary file 1 — Supporting Information [file CTM2-12-0-s002.docx]

**SUPPORTING INFORMATION**

**Prior SARS-CoV-2 infection balances immune responses triggered by four EMA-approved COVID-19 vaccines: an observational study**

Roberto Lozano-Rodríguez,^1,2,$^ Verónica Terrón-Arcos, ^1,2,$^ Karla Montalbán-Henández,^1,2^ José Carlos Casalvilla-Dueñas,^1,2^ Marta Bergón-Gutierrez,^1,2^ Alejandro Pascual-Iglesias,^1,2^ Jaime Valentín Quiroga, ^1,2^ Luis A. Aguirre, ^1,2^ Rebeca Pérez de Diego,^1^ Carmen Vela-Olmo,^3^ Lissette López-Morejón,^3^ Alejandro Martín-Quirós,^4^ Álvaro del Balzo-Castillo,^1,4^ María A. Peinado-Quesada,^4^ Miguel A. García-Garrido,^4^ Laura Gómez-Lage,^4^ Carmen Herrero-Benito,^4^ Irene Llorente-Fernández,^5^ Gema Martín-Miguel,^5^ Margarita Torrejon,^5^ Carolina Cubillos-Zapata,^6,7^ Carlos del Fresno,^1,2^ José Avendaño-Ortiz,^1,2,^**^*^** Eduardo López-Collazo,^1,2, 7,^**^*,^**^&^

^1^The Innate Immune Response Group, IdiPAZ, La Paz University Hospital, Madrid, Spain.

^2^Tumour Immunology Lab, IdiPAZ, La Paz University Hospital, Madrid, Spain.

^3^Eurofins-Ingenasa, Madrid, Spain.

^4^Emergency Department and Emergent Pathology Research Group, IdiPAZ La Paz University Hospital, Madrid, Spain.

^5^Intensive Care Unit, Hospital 12 de Octubre, Madrid, Spain.

^6^ Respiratory Diseases Group, IdiPAZ, La Paz University Hospital, Madrid, Spain.

^7^ Network Biomedical Research Center in Respiratory Diseases (CIBERES), Madrid, Spain.

^$^Equal contribution

^*^Corresponding authors Eduardo López-Collazo and José Avendaño-Ortiz.

Emails: [elopezc@salud.madrid.org](mailto:elopezc@salud.madrid.org) (E.L-C.) and [jose.avendano@idipaz.es](mailto:jose.avendano@idipaz.es) (J.A-O.)

^&^Leading contact E.L.-C.: [elopezc@salud.madrid.org](mailto:elopezc@salud.madrid.org)

**Table of contents**

[**Author Contributions** 3](#_Toc100249531)

[**Acknowledgements** 3](#_Toc100249532)

[**Ethical approval** 3](#_Toc100249533)

[**Competing interests** 3](#_Toc100249534)

[**Material and Methods** 4](#_Toc100249535)

[*Patient recruitment and sample collection* 4](#_Toc100249536)

[*PBMCs isolation, cryopreservation and storage procedure* 4](#_Toc100249537)

[*Cell culture* 5](#_Toc100249538)

[*Plasma collection* 5](#_Toc100249539)

[*Detection of anti-Spike RBD IgAs and IgGs and anti-Nucleocapsid IgG SARS-CoV-2 antibodies* 5](#_Toc100249540)

[*Detection of neutralization capacity of plasma against SARS-CoV-2 Spike antigen* 5](#_Toc100249541)

[*Specific Spike SARS-CoV-2 T cell proliferation assays and supernatant collection* 6](#_Toc100249542)

[*Supernatant soluble cytokine quantification* 6](#_Toc100249543)

[*Statistical analysis* 6](#_Toc100249544)

[*Study limitations* 7](#_Toc100249545)

[**Figure S1. Anti-RBD IgA levels triggered by ChAdOx1, Ad26.CoV2.S, mRNA-1273 and BNT162b2 in naïve individuals over time.** 8](#_Toc100249546)

[**Figure S2. SARS-CoV-2 specific CD8^+^ proliferation in COVID-19 naïve individuals classified by vaccination regimen and time since full vaccination.** 9](#_Toc100249547)

[**Figure S3. SARS-CoV-2 specific cytokine production** **in COVID-19 naïve individuals classified by vaccination regimen and time since full vaccination.** 10](#_Toc100249548)

[**Figure S4. Time curves of memory T cell subpopulations of Spike-specific proliferative cells in COVID-19 naïve individuals by vaccination regimen.** 11](#_Toc100249549)

[**Table S1. Baseline characteristics of SARS-CoV-2 naïve individuals included in the study.** 12](#_Toc100249550)

[**Table S2. Baseline characteristics of SARS-CoV-2 recovered individuals included in the study.** 13](#_Toc100249551)

[**Table S3. List of fluorochrome-conjugated monoclonal antibodies for T cell proliferation assay by flow cytometry analysis** 14](#_Toc100249552)

**Author Contributions**

ELC, JAO and RLR designed the study. ELC and JAO wrote the manuscript. AMQ, MAGG, ABC and MP recruited the participants and collected the samples. RLR, JAO, KMH, JCD, MBG, RPD and VT performed the analysis and the neutralizing antibodies quantification. RLR, JAO, JVQ and VT acquired the samples by FACS staining and analyzed the cellular immune response. ELC, CdF, RLR, JAO, API, CCZ and LAA discussed the results. RLR and CdF performed a critical review of the manuscript. All authors read and agreed to submit the manuscript for publication.

**Acknowledgements**

This study was supported by Fundación Uría. Authors thank to Carlos Castaneda for his collaboration. In memoriam of Norma López-Collazo.

**Ethical approval**

Informed consent was obtained from all participants in accordance with the hospital’s ethical standards and following the ethical guidelines of the 1975 Declaration of Helsinki. The study was authorized by the La Paz University Hospital Research Ethics Committee (PI-4100).

**Competing interests**

The authors have declared that no conflict of interest exists.

## **Material and Methods**

### *Patient recruitment and sample collection*

We designed a prospective observational analysis (Figure S1) with a total cohort of 433 fully vaccinated individuals with EMA-approved vaccines before August 8, 2021. Recruitment was performed from February 21, 2021 to October 21, 2021. Individuals were classified in two groups: no previously infected (naïve, n=292), and those with previous diagnosis of SARS-CoV-2 infection or not diagnosed but having detectable levels of anti-Nucleocapsid protein IgG levels determined by ELISA (recovered, n=141). Volunteers were subclassified according to days after full vaccination: Period 1, <90 (mean±SD, 57±31) days, Period 2, 90-150 (mean±SD, 119±13) days, Period 3, 150-210 (mean±SD, 163±22) days and Period 4 >210 (mean±SD, 229±12) days for COVID-19 naïve individuals and Period 1, <150 (mean±SD, 87±42) days and Period 2, >150 (mean±SD, 200±50) days for recovered individuals. All were asymptomatic for more than 14 days at the moment of blood drawing. Due to the descriptive characteristics of our study, there was no predefined sample size. All participants signed an informed consent and data were anonymized before study inclusion, and their details are summarized in **Supplementary Tables S1 and S2**.

### *PBMCs isolation, cryopreservation and storage procedure*

Peripheral blood mononuclear cells (PBMCs) from healthy volunteers vaccinated with the ChAdOx1, Ad26.CoV2.S, mRNA-1273, and BNT162b2 vaccines against SARS-CoV-2 were isolated from EDTA anticoagulant venous blood using Ficoll-Plus (GE Healthcare Bio-Sciences) solution according to the manufacturer’s instructions. PBMCs were washed twice with phosphate buffer saline (PBS) and counted using Trypan blue staining. A part of cells was resuspended in two aliquots of 6 ∙ 10^6^ cells in foetal bovine serum (FBS) containing 10% DMSO (Sigma-Aldrich). Then, aliquoted PBMCs were slowly frozen (-1 °C/minute) using a controlled-grade freezing device (Mr. Frosty, ThermoFisher Scientific) and stored for 24 hours at -80 ⁰C before storage in liquid nitrogen.

### *Cell culture*

Fresh and thawed PBMCs were cultured in RPMI 1640 medium containing 10% foetal bovine serum (FBS), 25 mM HEPES, 2 mM L-glutamine and 1% Penicillin and Streptomycin Mix (Gibco) before some stimulation to their activation or proliferation. PBMCs were cultured at 37 °C at 5% CO_2_ in a humidified incubator.

### *Plasma collection*

Plasma samples from healthy volunteers vaccinated with the ChAdOx1, Ad26.CoV2.S, mRNA-1273, and BNT162b2 vaccines against SARS-CoV-2 were obtained from EDTA anticoagulant venous blood using Ficoll-Plus (GE Healthcare Bio-Sciences) solution according to standard density gradient centrifugation method. Then, they were aliquoted and stored at -80 °C until use.

### *Detection of anti-Spike RBD IgAs and IgGs and anti-Nucleocapsid IgG SARS-CoV-2 antibodies*

For detection of specific antibodies IgA and IgG against the Spike protein of SARS-CoV-2, reserved plasma samples from healthy volunteers vaccinated with the ChAdOx1, Ad26.CoV2.S, mRNA-1273, and BNT162b2 vaccines against SARS-CoV-2 stored at -80 °C were thawed and centrifuged at 1000 relative centrifugal force for 30 minutes to remove particulates prior to use. The titer of IgA and IgG antibodies in plasma samples were performed by the bead-based multiplex assay, LEGENDplex SARS-CoV-2 Serological IgA and IgG Panels (1-plex, receptor binding domain (RBD) of Spike protein) (Biolegend) according to the manufacturer’s instructions. The titer of anti-Nucleocapsid antibodies in plasma samples were performed by INGEZIM^®^-NP-COVID 19 DR Eurofins-Ingenasa kit.

### *Detection of neutralization capacity of plasma against SARS-CoV-2 Spike antigen*

The neutralizing antibodies in plasma samples were performed by a competitive immunoassay of ACE2-conjugated beads, LEGENDplex SARS-CoV-2 Neut. Ab Assay (1-plex) according to the manufacturer’s instructions.

### *SARS-CoV-2 Spike-specific T cell proliferation assays and supernatant collection*

Fresh PBMCs from healthy volunteers vaccinated with the ChAdOx1, Ad26.CoV2.S, mRNA-1273, and BNT162b2 vaccines against SARS-CoV-2 isolated from EDTA anticoagulant venous blood using Ficoll-Plus (GE Healthcare Bio-Sciences) were washed twice with phosphate buffer saline (PBS) and counted using Trypan blue staining. Carboxyfluorescein succinimidyl ester (CFSE) was purchased from Thermo Fisher Scientific and used following the manufacturer’s protocol to assess T lymphocyte proliferation. After that, CFSE-labeled PBMCs were plated in RPMI 1640 medium containing 10% fetal bovine serum (FBS), 25 mM HEPES, 2 mM L-glutamine and 1% Penicillin and Streptomycin Mix (Gibco) in a 96-wells plate flat bottom (1,5∙10^6^ cells/well) and stimulated or not with Peptivator SARS-CoV-2 Prot_S (Miltenyi Biotec) for 5 days at 37 °C at 5% CO_2_. After proliferation assay, supernatants were collected, aliquoted and stored at -80 °C until use. Then, PBMCs were washed and stained with fluorochrome-conjugated antibodies to surface markers listed in **Supplementary Table 3**.

### *Supernatant soluble cytokine quantification*

Reserved and stored supernatants of PBMCs from healthy volunteers vaccinated with the ChAdOx1, Ad26.CoV2.S, mRNA-1273, and BNT162b2 vaccines against SARS-CoV-2, stimulated with Peptivator SARS-CoV-2 Prot_S (Miltenyi Biotec) for 5 days, were thawed. The concentration measurements of cytokines in supernatant samples were performed by the bead-based multiplex assay, LEGENDplex Human Essential Immune Response Panel (3-plex: IFN-γ, TNF-α and IL-6), according to the manufacturer’s instructions. Samples were acquired on a FACSCalibur flow cytometer (BD Biosciences) and data were analyzed using LEGENDplex (BioLegend) v.8 software.

### *Statistical analysis*

Data are presented as numbers, percentages, means, and standard deviations as indicated in the figure legends. Differences between groups were evaluated with the use of a chi-squared test for categorical variables; Student’s t-test for comparison of quantitative variables between two groups, and Kruskal-Wallis with Dunn's multiple comparisons test for comparisons of quantitative variables including more than two groups. Tukey multiple comparisons teste were performed to measure mixed-effects in time-course curves. P-values of less than 0.05 were considered to indicate statistical significance. All P-values are 2-sided, and 95% confidence intervals (95% CI) are also presented. Statistical analyses were conducted using Prism 8.0 (GraphPad) and SPSS version 23 (IBM) software.

### *Study limitations*

We have not a predefined sample number, we recruited the subjects without any type of limitation or bias, in order to have a representative picture of what we can find in the general population. However, subsequently this resulted in a reduced number of samples in some of the analysed groups. Another limitation is that our study it is not a longitudinal analysis following the same individuals over time, but that instead, different individuals are making up the different time points. The variation between individuals could impact the values measured at different time points.

**SUPPLEMENTARY FIGURES**

**Figure S1. Anti-RBD IgA levels triggered by ChAdOx1, Ad26.CoV2.S, mRNA-1273 and BNT162b2 in naïve individuals over time.**

The anti-Spike RBD IgA levels in ng/mL determined by cytometric bead assay in plasma from naïve individuals are shown. *, P<0.05; **, P<0.01 in Kruskal-Wallis with Dunn’s multiple comparisons post-hoc test for group comparisons. Data in time curves expressed as mean±SEM; AUC: Area under the curve; **, P<0.01; ***, P<0.001 in Tukeys’s multiple comparisons test for time curve comparison.


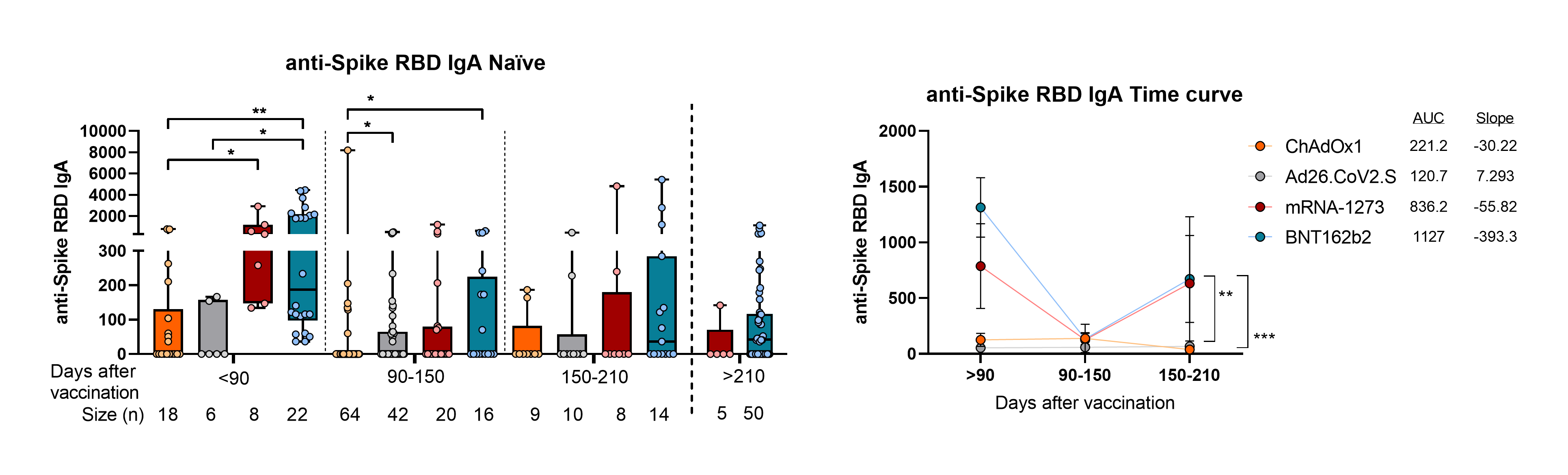


## **Figure S2. SARS-CoV-2 specific CD8^+^ proliferation in COVID-19 naïve individuals classified by vaccination regimen and time since full vaccination.**


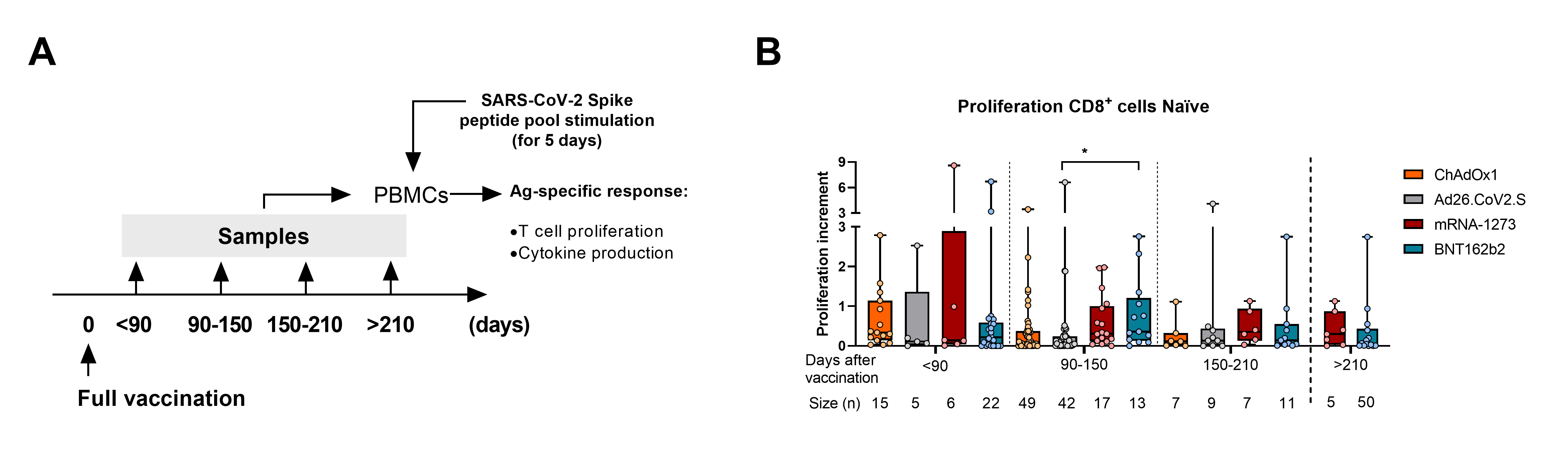


Non-previously infected individuals (naïve) were classified according to their EMA vaccine regimen and the time since full vaccination. (A) Procedure scheme. Peripheral blood cells were isolated from patients’ blood and stimulated with Spike SARS-CoV-2 peptide pool for five days. After that, cells were labelled and acquired by flow cytometry. (B) Proliferation increments of CD8^+^ cells after stimulation with SARS-CoV-2 peptide pool are shown. *, P<0.05 in Kruskal-Wallis with Dunn’s multiple comparisons post-hoc test.

## **Figure S3. SARS-CoV-2 specific cytokine production** **in COVID-19 naïve individuals classified by vaccination regimen and time since full vaccination.**


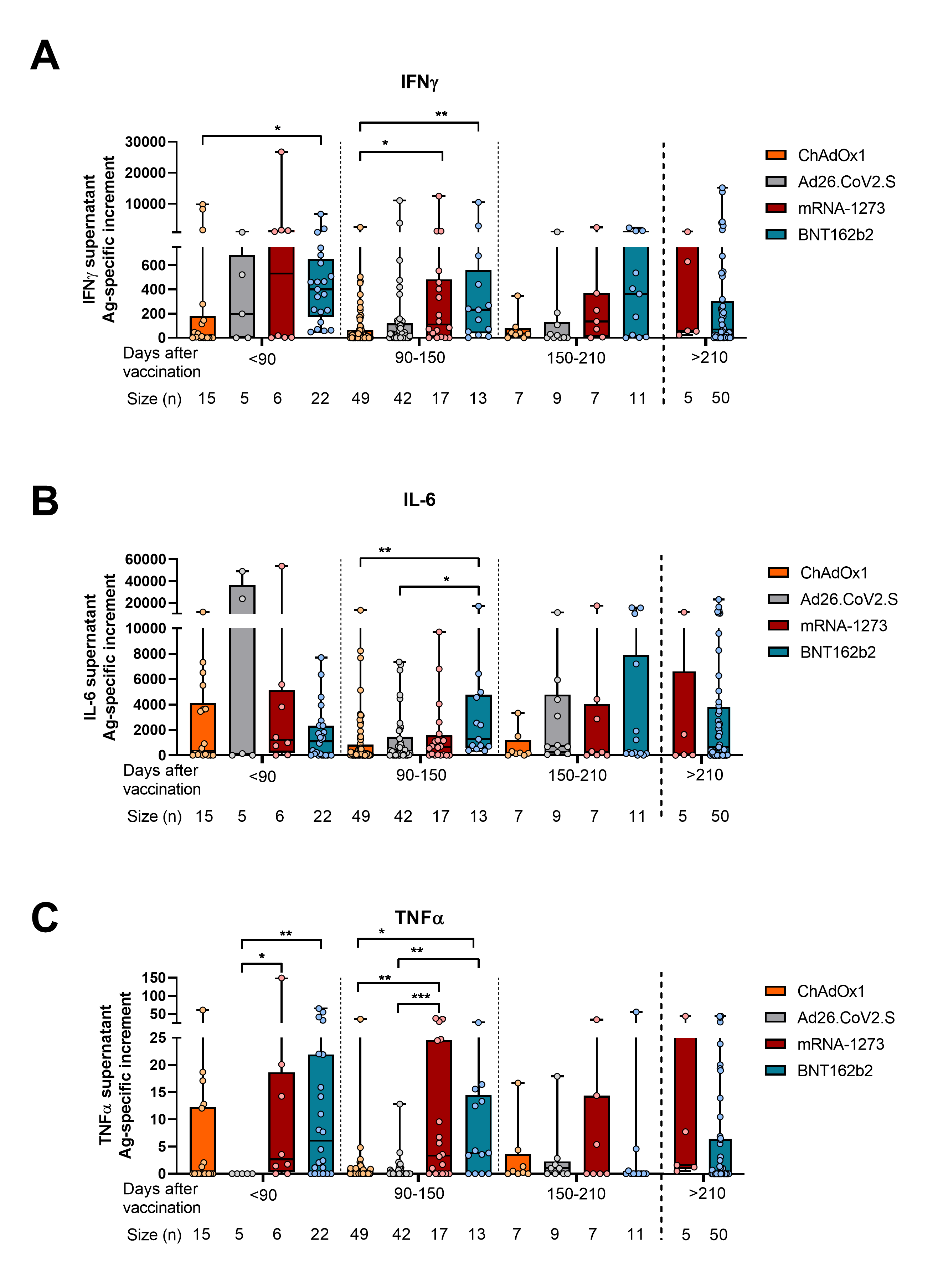


Non-previously infected individuals (naïve) were classified according to their EMA vaccine regimen and the time since full vaccination. Peripheral blood cells were isolated from patients’ blood and stimulated with Spike SARS-CoV-2 peptide pool for five days. After that, IFNγ (A), IL-6 (B) and TNFα (C) in the supernatant were quantified. Panels A, B and C show the cytokine production in pg/mL after stimulation with SARS-CoV-2 peptide pool. *, P<0.05; **, P<0.01 in Kruskal-Wallis with Dunn’s multiple comparisons post-hoc test.

## **Figure S4. Time curves of memory T cell subpopulations of Spike-specific proliferative cells in COVID-19 naïve individuals by vaccination regimen.**


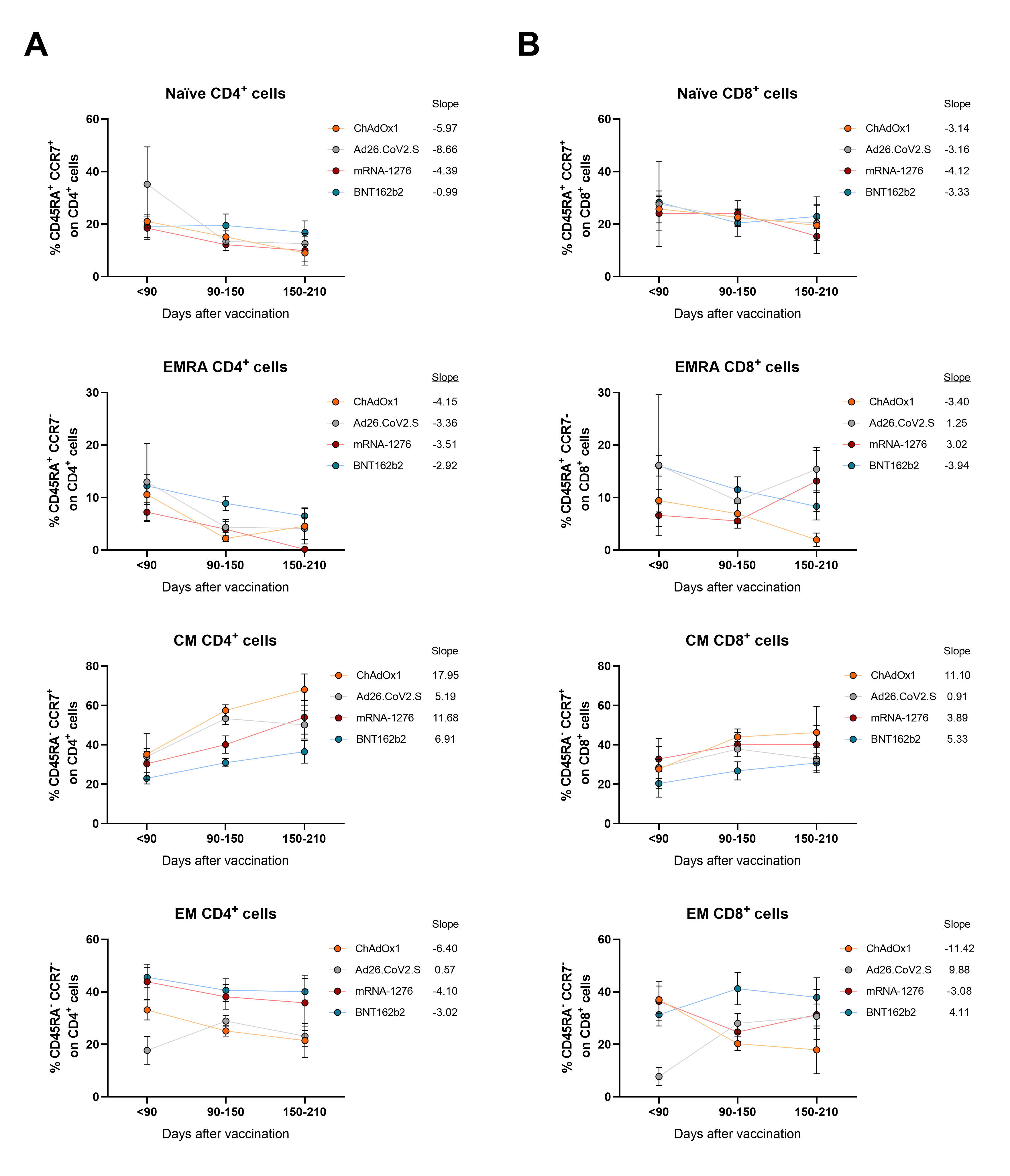


PBMCs were labelled with CFSE and stimulated with SARS-CoV-2 spike peptide pool for 5 days. CD4^+^ and CD8^+^ T cells were classified according to their proliferative response. Memory subpopulations were analyzed in proliferative cells (Naïve; central memory, CM; effector memory cells re-expressing CD45RA, EMRA; effector memory, EM). (A) The time curves of frequency of memory populations (Naïve, upper panel; EMRA, upper middle panel; CM, lower middle panel; EM, lower panel) in proliferative CD4^+^ cells from COVID-19 naïve individuals are shown. (B) The time curves of frequency of memory populations (Naïve, upper panel; EMRA, upper middle panel; CM, lower middle panel; EM, lower panel) in proliferative CD8+ cells from COVID-19 naïve individuals are shown. Data shown as mean±SEM. ChAdOx1, orange curve; Ad26.CoV.S, grey curve; mRNA-1273, red curve; BNT162b2 vaccine, blue curve; S, curve slope.

## **Table S1. Baseline characteristics of SARS-CoV-2 naïve individuals included in the study.**

| Days after full vaccination | <90 | | | | | 90-150 | | | | | 150-210 | | | | | >210 | | |
| --- | --- | --- | --- | --- | --- | --- | --- | --- | --- | --- | --- | --- | --- | --- | --- | --- | --- | --- |
| COVID-19 naïve | **AZ (n=18)** | **J (n=6)** | **M (n=8)** | **P (n=22)** | **P-value** | **AZ (n=64)** | **J (n=42)** | **M (n=20)** | **P (n=16)** | **P-value** | **AZ (n=9)** | **J (n=10)** | **M (n=8)** | **P (n=14)** | **P-value** | **M (n=5)** | **P (n=50)** | **P-value** |
| Age (years) | 58.3±13.2 | 51.8±10.5 | 44.0±16.8 | 45.0±12.3 | *, 0.010 | 52.0±13.4 | 48.4±6.2 | 46.6±14.8 | 56.9±10.8 | 0.053 | 51.6±13.5 | 57.1±9.9 | 56.3±15.4 | 46.4±18.3 | 0.317 | 34.2±14.8 | 40.9±11.8 | 0.133 |
| Sex (Male, %) | 8 (44.44) | 4 (66.7) | 4 (50.0) | 11 (50.00) | 0.828 | 25 (39.0) | 16 (38.1) | 7 (35.0) | 6 (37.5) | 0.991 | 3 (33.3) | 2 (20.0) | 1 (14.3) | 4 (28.6) | 0.743 | 0 (0.0) | 12 (24.0) | 0.215 |
| Coexisting disorder – n (%) |  |  |  |  |  |  |  |  |  |  |  |  |  |  |  |  |  |  |
| Hypertension | 4 (28.6) | 2 (33.3) | 1 (12.5) | 0 (0.0) | 0.077 | 6 (9.4) | 4 (9.5) | 3 (15.0) | 7 (43.8) | *, 0.011 | 2 (22.2) | 2 (20.0) | 2 (25.0) | 0 (0.0) | 0.292 | 0 (0.0) | 1 (2.0) | 0.750 |
| Diabetes Mellitus | 2 (11.1) | 0 (0.0) | 0 (0.0) | 0 (0.0) | 0.245 | 4 (6.3) | 1 (2.4) | 0 (0.0) | 0 (0.0) | 0.411 | 0 (0.0) | 0 (0.0) | 0 (0.0) | 0 (0.0) | >0.999 | 0 (0.0) | 0 (0.0) | >0.999 |
| Obesity | 3 (16.7) | 0 (0.0) | 1 (12.5) | 2 (9.1) | 0.701 | 2 (3.1) | 1 (2.4) | 1 (5.0) | 3 (18.8) | 0.056 | 0 (0.0) | 0 (0.0) | 0 (0.0) | 1 (7.14) | 0.577 | 0 (0.0) | 3 (6.0) | 0.573 |
| Oncologic Disease History | 1 (5.6) | 0 (0.0) | 0 (0.0) | 0 (0.0) | 0.565 | 4 (6.3) | 0 (0.0) | 2 (10.0) | 2 (12.5) | 0.196 | 0 (0.0) | 0 (0.0) | 2 (25.0) | 1 (7.14) | 0.159 | 0 (0.0) | 1 (2.0) | 0.750 |
| Autoimmune Disease | 2 (11.1) | 0 (0.0) | 2 (25.0) | 0 (0.0) | 0.102 | 1 (1.6) | 0 (0.0) | 1 (5.0) | 0 (0.0) | 0.441 | 0 (0.0) | 0 (0.0) | 0 (0.0) | 2 (14.3) | 0.256 | 0 (0.0) | 4 (8.0) | 0.511 |
| Renal Disease | 0 (0.0) | 0 (0.0) | 0 (0.0) | 0 (0.0) | >0.99 | 0 (0.0) | 0 (0.0) | 0 (0.0) | 1 (6.3) | *, 0.048 | 0 (0.0) | 0 (0.0) | 0 (0.0) | 1 (7.14) | 0.577 | 0 (0.0) | 0 (0.0) | >0.999 |

Data are expressed as mean±SD or number (percentage). AZ, AstraZeneca for ChAdOx1 vaccine; J, Janssen for Ad26.CoV2.S vaccine; M, Moderna for mRNA-1273; P, Pfizer for BNT162b2 vaccine.

P-values for One-way ANOVA for four groups comparison or Man Whitney for two group of quantitative variables and Chi-square for comparison of qualitative variables test are shown.

## **Table S2. Baseline characteristics of SARS-CoV-2 recovered individuals included in the study.**

| Days after vaccination | <150 | | | | | | | >150 | | | | |
| --- | --- | --- | --- | --- | --- | --- | --- | --- | --- | --- | --- | --- |
| COVID-19-recovered | **AZ 1x (n=7)** | **AZ 2x (n=14)** | **J 1x (n=14)** | **M 1x (n=7)** | **P 1x (n=6)** | **P 2x (n=24)** | **P-value** | **AZ 1x (n=27)** | **J 1x (n=5)** | **M 1x (n=3)** | **P 2x (n=34)** | **P-value** |
| Age (years) | 46.26±14.40 | 52.00±15.37 | 47.07±6.23 | 32.88±14.26 | 40.80±11.45 | 45.04±14.54 | 0.0796 | 48.46±12.93 | 48.57±7.44 | 28.25±7.41 | 37.88±8.00 | ***, 0.0006 |
| Sex (Male, %) | 3 (42.86) | 4 (28.57) | 7 (50.00) | 5 (71.43) | 2 (33.33) | 12 (50.00) | 0.5216 | 10 (37.04) | 4 (80.00) | 0 (0.00) | 8 (23.53) | *, 0.0410 |
| Coexisting disorder – n (%) |  |  |  |  |  |  |  |  |  |  |  |  |
| Hypertension | 1 (14.29) | 5 (35.71) | 1 (7.14) | 0 (0.00) | 0 (0.00) | 4 (16.67) | 0.1786 | 3 (11.11) | 0 (0.00) | 0 (0.00) | 0 (0.00) | 0.4568 |
| Diabetes Mellitus | 0 (0.00) | 0 (0.00) | 0 (0.00) | 0 (0.00) | 0 (0.00) | 2 (8.33) | 0.5212 | 0 (0.00) | 0 (0.00) | 0 (0.00) | 0 (0.00) | >0.9999 |
| Obesity | 1 (14.29) | 2 (14.29) | 3 (21.43) | 1 (14.29) | 1 (33.33) | 1 (4.17) | 0.7429 | 1 (3.70) | 0 (0.00) | 0 (0.00) | 0 (0.00) | 0.6643 |
| Oncologic Disease History | 1 (14.29) | 0 (0.00) | 0 (0.00) | 0 (0.00) | 0 (0.00) | 0 (0.00) | 0.0887 | 0 (0.00) | 0 (0.00) | 0 (0.00) | 0 (0.00) | >0.9999 |
| Autoimmune Disease | 0 (0.00) | 0 (0.00) | 0 (0.00) | 2 (28.57) | 2 (33.33) | 2 (8.33) | *, 0.0336 | 1 (3.70) | 0 (0.00) | 0 (0.00) | 2 (5.88) | 0.9003 |
| Renal Disease | 0 (0.00) | 0 (0.00) | 0 (0.00) | 0 (0.00) | 0 (0.00) | 1 (4.17) | 0.8394 | 0 (0.00) | 0 (0.00) | 0 (0.00) | 0 (0.00) | >0.9999 |

Data are expressed as mean±SD or number (percentage). AZ, AstraZeneca for ChAdOx1 vaccine; J, Janssen for Ad26.CoV2.S vaccine; M, Moderna for mRNA-1273; P, Pfizer for BNT162b2 vaccine. 1x refers to one shot-based vaccination regimes and 2x refers to two shot-based vaccination regimes.

P-values for One-way ANOVA for four groups comparison or Mann-Whitney for two group of quantitative variables and Chi-square for comparison of qualitative variables test are shown.

## **Table S3. List of fluorochrome-conjugated monoclonal antibodies for T cell proliferation assay by flow cytometry analysis**

| Marker | Fluorochrome | Source | Clone | Reference |
| --- | --- | --- | --- | --- |
| CD3 | BV510 | Biolegend | OKT3 | Cat# 317332 |
| CD4 | cFluor-YG584 | Cytek Biosciences | SK3 | Cat# R7-20042 |
| CD8 | BUV805 | BD | SK1 | Cat# 612889 |
| CD45 | PerCP | Biolegend | 2D1 | Cat# 368506 |
| CD45RA | BUV395 | BD | 5H9 | Cat# 740315 |
| CD62L | BUV615 | BD | SK11 | Cat# 751364 |
| CD28 | BV650 | Biolegend | CD28.2 | Cat# 302946 |
| CCR7 | BV421 | Biolegend | G043H7 | Car# 353208 |
